# Endothelial clock regulates retinal angiogenesis and ganglion cell function

**DOI:** 10.1007/s10456-025-10018-4

**Published:** 2025-11-15

**Authors:** Vijay K. Jidigam, Madison B. Kirby, Joshua Gallop, Brianna M. Shimandle, Dhwani Parsana, Minzhong Yu, Richard A. Lang, Sujata Rao

**Affiliations:** 1 Department of Ophthalmic Research, Cole Eye Institute, Cleveland Clinic, 9500 Euclid Avenue, Cleveland, OH 44195 USA; 2 Division of Developmental Biology, Cincinnati Children’s Hospital, Cincinnati, OH USA; 3 Department of Ophthalmology, College of Medicine, University of Cincinnati, Cincinnati, OH USA; 4 Department of Ophthalmology, Cleveland Clinic Lerner College of Medicine of Case Western Reserve University, Cleveland, OH 44195 USA

**Keywords:** Endothelial clock, Angiogenesis, ERG, PhNR, Cell cycle, RGCs

## Abstract

Angiogenesis, the formation of new blood vessels from existing ones, is essential for physiological and pathological processes such as wound healing, organ development, and tumor growth. It is a tightly regulated process influenced by both intrinsic and extrinsic factors. Emerging evidence shows a connection between biological clocks that regulate physiological rhythms and angiogenesis through the modulation of angiogenic factors like vascular growth factor (Vegfa). Thus, the clock system can directly modulate the timing and efficiency of angiogenic processes. This study aimed to investigate the role of key circadian clock genes, Bmal1 and Per2, in retinal angiogenesis. Endothelial cell-specific deletion of these genes significantly impairs vessel growth, although distinct phenotypic differences emerge between the two knockout models as angiogenesis progresses. RNA-sequencing (RNA-seq) analysis of retinal endothelial cells reveals that circadian clocks predominantly influence the expression of genes involved in cell proliferation. Notably, vascular endothelial cell (VEC) proliferation is diurnally regulated and is disrupted in Bmal1 knockout animals, leading to an increase in the number of Brn3a-positive retinal ganglion cells (RGCs). These alterations are further associated with compromised retinal circuitry and function. Thus, this study uncovers critical roles for Bmal1 and Per2 in regulating retinal angiogenesis, emphasizing the importance of circadian control of cell proliferation in vascular development and retinal function.

**Supplementary Information:**

The online version contains supplementary material available at 10.1007/s10456-025-10018-4.

## Introduction

Circadian rhythms are daily oscillations in biological functions optimized for specific times of day, enabling organisms to adapt to their external environment. Disruptions to these rhythms, whether due to genetic or behavioral factors, can lead to cardiovascular and metabolic dysfunction. Cell and tissue-specific circadian rhythms are generated by a complex transcriptional-translational feedback loop of master clock genes, creating a self-sustaining autoregulatory cycle of approximately 24 h in their transcripts and proteins. This feedback loop is mediated by transcriptional activators, Brain and Muscle ARNT-Like 1 (BMAL1) and Circadian Locomotor Output Cycles Kaput (CLOCK), as well as transcriptional repressors, Cryptochrome (CRY) and Period (PER), and interlocking loops involving the ROR, REV-ERB, and DEC proteins [1–4]. Transcription factors BMAL1 and CLOCK form a heterodimer that promotes transcription of clock control genes (CCGs), including PER and CRY. Accumulation of PER and CRY proteins, in turn, represses transcriptional activity of BMAL1/CLOCK complex, reducing expression of CCGs [5–7]. BMAL1 regulates a wide range of downstream pathways, including those involved in cell cycle control, oxidative stress response, metabolism, and angiogenesis [8–12]. In endothelial cells, Bmal1 influences vascular homeostasis, nitric oxide signaling, and cellular proliferation [13, 14]. Deletion of Bmal1 or Per2 can lead to overlapping phenotypes due to disruption of the entire clock system or compensatory mechanisms within the circadian network [15]. Bmal1 is unique among the core clock genes in that its disruption alone leads to arrhythmicity. Hence, it is a critical element of the manipulation of molecular clocks in vitro and in vivo.

Circadian clocks are integral to vascular physiology, orchestrating daily rhythms in cardiovascular function to align tissue perfusion with fluctuating metabolic and functional demands. Both vascular contractibility and pressure exhibit robust 24-h oscillations under circadian control. Notably, the incidence of cardiovascular events such as stroke peaks during the early hours of the morning, further underscoring the temporal regulation of vascular function. While the role of the vascular circadian clock in adult homeostasis is well established, its function during development remains poorly understood [16, 17].

In rodents, retinal vascular development occurs postnatally through angiogenesis, resulting in the development of three spatially distinct vascular plexuses. The superficial vascular layer adjacent to the ganglion cell layer develops first, followed by the deep and intermediate layers within the outer and inner plexiform layers, respectively [18, 19]. Thus, retinal angiogenesis is spatially and temporally regulated, requiring synchronized proliferation and migration of endothelial cells. A crucial function of the circadian clock is to align cellular activity with environmental cues [20]. This raises the possibility that the circadian clock may similarly regulate retinal angiogenesis by coordinating vascular endothelial cell (VEC) responses to angiogenic signals, thereby impacting the timing and pattern of vascular growth. Our data show that loss of Bmal1 and Per2 from the VEC results in reduced retinal VEC proliferation and slower vessel growth. Furthermore, transcriptomic analysis of enriched endothelial cells from Bmal1 conditional knockout (Bmal1; CKO) animals indicates that Bmal1 regulates cell cycle and angiogenesis-related pathways, suggesting that the circadian clock plays an essential role in vascular development. Additionally, our data shows a temporal regulation of retinal endothelial cell proliferation, with a majority of cells in S-phase of the cell cycle during the night, when mice are most active. In contrast, retinal VECs in the Bmal1; CKO animals exhibit reduced proliferation, regardless of the time of day. Our findings reveal that Bmal1 deletion disrupts both vascular development and retinal ganglion cell function, underscoring the critical role of circadian regulation in retinal health.

## Materials and methods

### Mouse strains

Transgenic strains used in this study are as follows: Bmal1^FL/FL^ (Jax stock no. 007668), B6.Cg-*Gt(ROSA)26Sor*^*tm14(CAG−tdTomato)Hze*^/J (Jax stock no. 007914, Ai14 reporter mice, also referred to as tdTomato in this manuscript), Per2^tm1a (EUCOMM)Hmgu^ [21], Per2^tm1Drw^ (Jax stock no. 010492) and Pdgf-icreER [18, 22]. For Cre induction, tamoxifen (T6648-1G, Sigma, St. Louis, MO, USA) dissolved in canola oil was administered intraperitoneally at a dosage of 15 μg/g body weight daily for three days, starting from postnatal day 0.5 (P0.5) until P2.5. Both control (Bmal1^FL/FL^, Per2^FL/FL^, tdTomato^FL/+^;Pdgf-icreER), and mutant (Bmal1^FL/FL^;Pdgf-icreER, Per2^FL/FL^;Pdgf-icreER, Bmal1^FL/FL^; tdTomato^FL/+^;Pdgf-icreER) pups were injected with tamoxifen to ensure consistent treatment across groups. All wild-type (WT) animals and the transgenic animals used in the study were derived from C57BL/6 J background. All animals were maintained in a light/dark (14:10 h) cycle and fed ad libitum with normal chow. Animal use protocol was approved by the Institutional Animal Care and Use Committee at Cleveland Clinic (IACUC), and all procedures followed the Association for Research in Vision and Ophthalmology (ARVO) guidelines for the use of animals in vision research.

### Tissue processing and Immunofluorescence staining

For cryosectioning, enucleated eyes were collected at noon (ZT6), fixed in 4% paraformaldehyde (PFA) for 90 min on ice, followed by overnight cryoprotection in 15% and 30% sucrose, respectively, at 4 °C. Cryoprotected eyes were embedded in optimal cutting temperature (OCT) compound and oriented dorsoventrally as described [23]. Sections were rinsed in phosphate-buffered saline (PBS), washed 2 times in 0.1% Triton X-100, permeabilized for five minutes with 1% Triton X-100, and blocked for one hour at room temperature (RT) in PBS containing 3% bovine serum albumin (BSA) and 0.1% Triton X-100. Cryosections were subsequently incubated overnight with the specified primary antibodies: Sox9 (1:100) (AB5535, Millipore, Temecula, CA, USA), GFAP (1:250) (NBP1-05197AF488, Novus Biologicals, Minneapolis, USA), Brn3a (1:250) (MAB1585, Sigma-Aldrich, St. Louis, MO, USA), Caspase-3 (1:100) (BD Pharmingen, San Jose, CA, USA), Bmal1 (1:100) (Abcam, AB3350, Waltham, MA, USA), and conjugated Alexa 488-Isolectin-IB4 (1:500) (I21411, ThermoFisher Scientific, USA). Appropriate Alexa Fluor conjugated secondary antibodies were used for labeling (ThermoFisher Scientific, USA). The sections were mounted in Fluoro-Gel mounting medium (201,009, Hatfield, PA). Only sections through the optic nerve were used for staining and quantification.

For whole mounts, retinas were dissected and fixed in 4% PFA for 1–2 h on ice, washed 3 times in PBS for 10 min, permeabilized for 1 h using 1% Triton X-100, and blocked in 0.03% Triton X-100 with 3% BSA in PBS for 1 h at RT. Retinas were incubated with Alexa 488-Isolectin IB4 (1:200) for 16–48 h at 4 °C. Appropriate Alexa Fluor conjugated secondary antibodies were used for labeling (ThermoFisher Scientific, USA). The retinas were mounted in Fluoro-Gel mounting medium (201,009, Hatfield, PA). Retinas were generally collected at noon (ZT6), unless specified otherwise. Imaging was done using a Leica laser scanning confocal microscope as described [24]. For Brn3a analysis at P7.5, Brn3a-positive cells were counted in regions located 400 μm away from the optic nerve head on either side, within a boxed region of 100 μm × 100 μm. Total counts from both sides were averaged and recorded as a single value per eye from different animals. At P24.5, Brn3a-positive cells were quantified across the entire retinal cryosection, and total counts were compared between groups, with each point representing one eye from one animal. For branch point and caspase-3 counts, an area of 581.25 µm X 581.25 µm from each quadrant (dorsal, ventral, anterior, posterior), closer to the angiogenic front, was used for quantitation. Branch points from all four quadrants were averaged to represent the data for a single eye. Vascular front migration is a ratio of the length from the optic nerve head to the periphery and the distance of the angiogenic front from the optic nerve.

### Retinal endothelial cell isolation using CD31 beads

Following enucleation, retinas were dissected from P7.5 pups in ice-cold Dulbecco’s Modified Eagle Medium (DMEM). Retinal dissociation was performed in DMEM containing 10 mg/ml collagenase A (10,103,578,001, Roche, USA) and 3 U/ml DNase I (D5025, Sigma, St. Louis, MO, USA) at 37 °C for 20 min, with pipetting every 10 min. The cell suspension was then passed through a 70 µm cell strainer (Fisher Scientific, 22,363,548) and centrifuged at 1870 g for 6–8 min. The resulting cell suspension was used to isolate enriched endothelial cells using CD31 magnetic beads, following the manufacturer’s protocol (Miltenyi Biotec, 130–097-418, North Rhine-Westphalia, Germany). Enriched endothelial cells were subsequently subjected to RNA isolation (Qiagen RNeasy MiniPrep Kit, Qiagen, Germantown, MD, USA) and cDNA synthesis (Verso cDNA Synthesis Kit) according to the manufacturer’s protocol. Validation experiments for gene expression based on RNA-sequencing data were performed on enriched endothelial cells.

### Fluorescence-activated cell sorting (FACs) of retinal endothelial cells

Retinal endothelial cells were isolated using fluorescence-activated cell sorting (FACS) as previously described [11]. Endothelial cells were FAC-sorted from P7.5 retinas of tdTomato^FL/+^;Pdgf-icreER (control) or Bmal1^FL/FL^;tdTomato^FL/+^;Pdgf-icreER (mutant) animals. All endothelial cells were labeled with GFP, which is expressed from the Pdgf-icreER transgene, as well as tdTomato expressed by cre-mediated recombination. For the transcriptomic analysis, GFP/Tomato double-positive sorted endothelial cells were used (Supplementary Fig. 4).

### RNA-seq analysis

RNA integrity was evaluated using an Agilent Bioanalyzer, and samples with an RNA Integrity Number (RIN) > 8.5 were used for RNA sequencing. Total RNA from sorted retinal endothelial cells (~ 500 pg/µl), isolated from Bmal1^FL/FL^;tdTomato^FL/+^;Pdgf-icreER (*n* = 3) and tdTomato^FL/+^;Pdgf-icreER (*n* = 3) animals, was amplified using the Smart v4 kit (634,888, Takara Bio USA, Inc. Mountain View, CA, USA). Libraries were prepared with the Nextera XT Library Preparation kit (15,032,355, San Diego, CA, USA) and sequenced for poly-A RNAs by an external NGS facility (Novogene). RNA-seq was performed on a HiSeq2000 sequencer (Illumina), generating approximately 100 million paired-end reads of 150 bp per sample. Fastq files were quality checked with FastQC, trimmed with Trimmomatic, and aligned to the reference mm10 mouse genome (Ensembl GRCm38 v87) using Bowtie. Counts were generated with Feature Counts, and differential expression analysis was conducted with DESeq2 (log2FC > 1.0, *P* < 0.05, FDR < 0.05). FastQC analysis indicated that the majority of reads across all samples had a Phred quality score above 30, demonstrating high base-calling accuracy. Each biological replicate yielded between 150 million and approximately 250 million reads. Over 70% of reads were uniquely mapped to the mouse reference genome, indicating high mapping efficiency. Importantly, all samples displayed similar sequencing quality metrics, supporting the consistency and comparability of the dataset (Supplementary Table 2). The alignment resulted in a total of 55,488 gene counts. All RNA-seq data processing and downstream analyses were performed using R, with subsequent downstream analysis done via Ingenuity Pathway Analysis (IPA) (v24.0.1) and InstantClue (v 0.11.1).

### Quantitative PCR

For quantitative PCR analysis, total RNA was extracted from neural retina using a RNeasy Mini Kit (Qiagen, Hilden, Germany) according to the manufacturer’s instructions. 250 ng of DNAse-treated RNA was reverse-transcribed using a Verso Enzyme cDNA synthesis kit (Thermo Fisher Scientific, Waltham, MA, USA). cDNA amplification was performed with gene-specific primers listed in Supplementary Table 1. Real-time PCR was performed using Radiant SYBR Green Low-ROX qPCR mix (Alkali Scientific, Pompano Beach, FL, USA) on a QuantStudio 3 Real-Time PCR System (Bio-Rad). Each assay was run in biological duplicates or triplicates, with two to three technical replicates per assay. β-actin served as the endogenous control for relative quantification of gene expression levels between samples using the comparative Ct method [25]. Subsequently, the 2^ΔΔ*Ct*^ method was used to evaluate the relative expression level (fold change).

### ddPCR

Reaction mixtures were prepared with primers (250 nM), template DNA (250 ng), and QX200™ ddPCR™ EvaGreen Supermix (Bio-Rad: 1,864,034) in a volume of 20ul, according to the manufacturer’s instructions. Droplet generation and emulsified sample transfer to PCR plates were performed using the Bio-Rad QX200™ Droplet Generator according to the manufacturer’s protocol. The ddPCR plate was sealed using a foil heat seal (Bio-Rad: 1,814,040) and the PX1™ PCR Plate Sealer (Bio-Rad: 181–4000). For transcript quantification, the QX200 Droplet Digital PCR (ddPCR™) System (Bio-Rad) was utilized. The absolute quantity of DNA per sample (copies/µL) was analyzed using QuantaSoft software (v.1.7.4.097) and QuantaSoft Analysis Pro (v.1.0) for data analysis.

### EdU labeling

To label proliferating cells, 5-ethynyl-2′-deoxyuridine (EdU; 100 µg/g body weight) was administered via intraperitoneal injection at postnatal days P4.5, P7.5, P8.5, and P9.5. Eyes were harvested two hours after injection. EdU staining was performed according to the manufacturer’s protocol using the Click-iT EdU Imaging Kit (Invitrogen, C10340, Oregon, USA). Endothelial cell proliferation was assessed by counting cells double-labeled with isolectin and EdU. For dark-rearing (DD) experiments, pregnant dams were transferred to a dark room shortly before delivery, and the pups were maintained in constant darkness until P7.5. On P7.5, EdU was injected at circadian time (CT) 12:00 pm using a head-mounted red light to minimize light exposure. Pups remained in darkness for two hours post-injection before enucleation.

### EdU normalization by vessel density

To account for differences in vessel density between day and night, EdU + cell counts were normalized to the corresponding vessel density for each sample. Vessel density was measured as the total vascular area within a region of 513 × 417 pixels. Normalized EdU counts were obtained as a ratio of total EdU + cells and vessel density. Cell counts were done with QuPath software and Angiotool for vessel density counts [26, 27].

### Photopic negative response and electroretinograms

Mice were anesthetized using a combination of ketamine (80 mg/kg) and xylazine (16 mg/kg), diluted in saline solution. To induce mydriasis, eye drops containing 1% mydriacyl, 1% cyclopentolate HCl, and 2.5% phenylephrine HCl were applied, and 0.5% proparacaine HCl was used to locally anesthetize the corneal surface. During electroretinogram (ERG) recording, animals were kept warm on a temperature-controlled heating pad. ERG signals were captured from the corneal surface using a stainless-steel electrode, which was placed in contact with the cornea through a thin layer of 0.7% methylcellulose. Subcutaneous needle electrodes in the cheek and tail served as reference and ground electrodes, respectively. White light flashes, ascending in intensity from 0.4 to 2.2 log cd s/m^2^, were deliveredagainst a green light background at 40 cd/m^2^ for light adaptation. The ERG signals were amplified (0.3–300 Hz), digitized at 1000 Hz, and averaged over 25 trials using the UTAS Bigshot Electrophysiology System (LKC Technologies, Gaithersburg, MD, USA), with a 60 Hz notch filter applied. The b-wave amplitude was measured from the trough of the a-wave, and the photopic negative response (PhNR) amplitude was quantified from the baseline to its trough. The ratio of PhNR amplitude to b-wave amplitude was calculated to assess the RGC’s function.

### Statistical analysis

Statistical analyses were conducted using GraphPad Prism 9.0 (La Jolla, CA, USA) and SPSS Statistics Subscription (Version 29). For PhNR analysis, repeated measures ANOVA was employed to detect overall differences in the PhNR/b-wave amplitude ratio between mutant and control groups across five stimulus luminance levels. The dependent variable was the PhNR/b-wave ratio. Stimulus luminance (five levels) served as the within-subject fixed factor, while genotype (mutant vs. control) was the between-subject fixed factor. This design enabled evaluation of both the main effect of genotype and the interaction between genotype and luminance. Dark-adapted and light-adapted ERG component amplitudes were similarly evaluated across four to ten stimulus luminance levels. Assumptions of normality and sphericity were tested before analysis. Mauchly’s test was used to assess sphericity; if violated, the Greenhouse–Geisser correction was applied. Post hoc pairwise comparisons with Bonferroni correction were performed to identify specific luminance levels at which significant differences between groups occurred. Additional analyses using *t*-tests and one-way ANOVA were conducted where appropriate. A significance threshold of α = 0.05 was applied for all statistical tests.

## Results

### Endothelial loss of Bmal1 impairs retinal vascular development

**Fig. 1:**
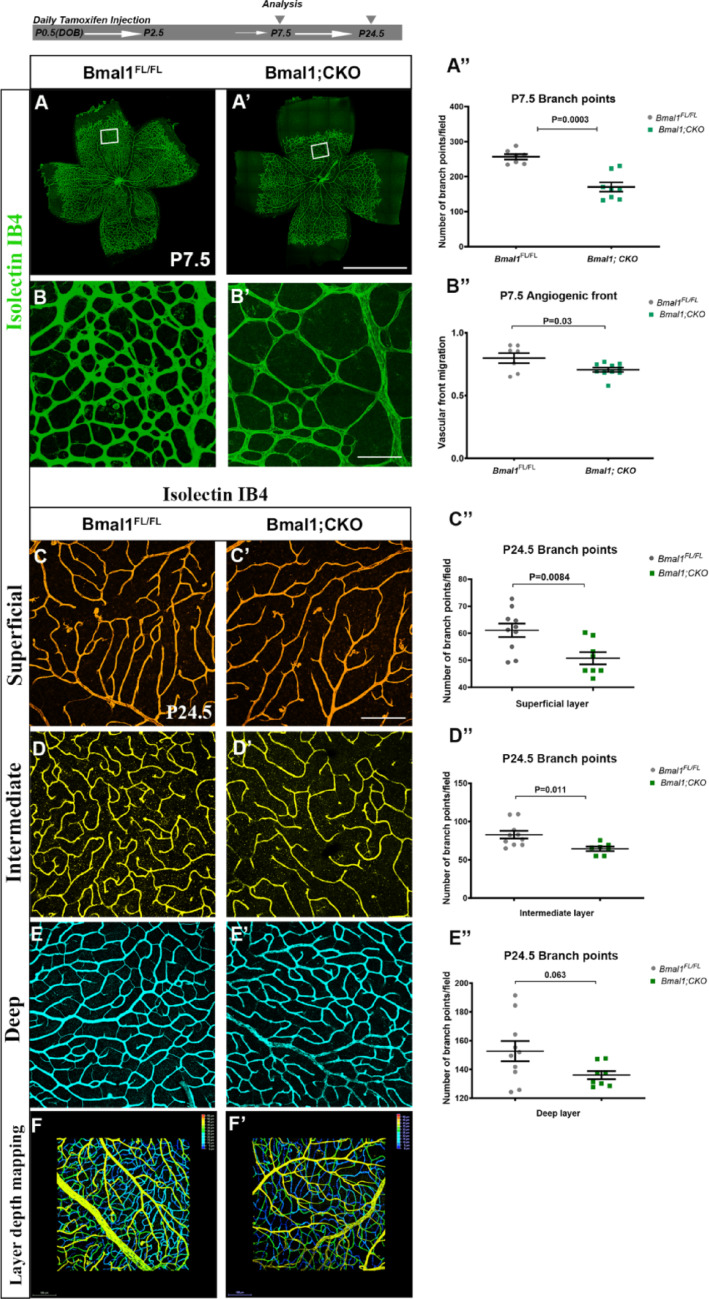
Endothelial-specific Bmal1 deletion leads to defects in retinal angiogenesis: For endothelial-specific Bmal1 deletion, pups were injected with tamoxifen from the day of birth (P0.5) through P2.5, and eyes were harvested at different ages as indicated. (**A**, **A**′) Retinal flat mounts labeled with Isolectin-IB4 to visualize retinal vasculature at P7.5. (**B**, **B**′) Higher magnification images of the boxed regions highlighting differences in vessel density between groups. (**A**′′, **B**′′) Bmal1; CKO pups show a significant reduction in vascular density (A′′) in the remodeling area, along with delayed angiogenic front migration (B′′) compared to Bmal1^FL/FL^ controls. Representative layer-specific flat mount images of Isolectin-stained retinas from Bmal1^FL/FL^ (**C**, **D**, **E**) and Bmal1;CKO (**C**′, **D**′, **E**′) animals. (**C**′′, **D**′′, **E**′′) Quantification of vessel density in superficial, intermediate, and deep layers. Note that only superficial and intermediate layers show significant density reduction in Bmal1;CKO when compared to Bmal1^FL/FL^ controls at P24.5. (F, F′) Depth-coded images of all three vascular layers, with yellow indicating the superficial layer, green the intermediate layer, and blue the deep layer. Statistical significance was calculated using Student’s t-test, and each data point represents a single eye. Error bars represent ± SEM, with *n* = 7–10. Scale bars: 800 µm (flat mounts, A′), 50 µm (field images, B′ and C′), and 100 µm (F and F′), respectively

Bmal1 is a critical component of the circadian clock; therefore, deletion of Bmal1 is often used as a common strategy to disrupt the clock function. Bmal1 was deleted from endothelial cells postnatally using the Pdgf-icreER transgenic animals as previously described [11]. At P7.5, loss of Bmal1 results in reduced vessel density (Fig. 1A′, B′, A′′) compared to the control retina (Fig. 1A, B, A′′, F, F′). Vascular density is similar between tamoxifen injected Pdgf-icreER and Bmal1^FL/FL^ animals; hence, Bmal1^FL/FL^ animals served as the control animals in all experiments. Furthermore, there is no difference in vascular density between the tamoxifen injected and uninjected animals thereby eliminating the possibility of nonspecific Cre expression without tamoxifen induction (Supplementary Fig. 1). In addition to reduced vessel density, the distance from the optic nerve head to the angiogenic front is significantly shorter in Bmal1 mutant retinas compared to controls (Fig. 1A, A′, B′′), suggesting that vessel migration is also affected. As angiogenesis progresses, angiogenic sprouts emerge from the superficial layer and penetrate the deeper layer, where they continue to proliferate, to give rise to the deeper vascular plexus. In control retina, as expected at P7.5, several angiogenic sprouts are detected, but in Bmal1;CKO animals, there is a complete absence of sprouts, indicating delayed vascular growth in the mutant retina (Supplementary Fig. 6, Supplementary Video 1, Supplementary Video 2). The superficial layer is completed by P7.5 and by P24.5; all three vascular layers, namely the superficial, intermediate, and deep layers, are fully formed. To assess whether loss of Bmal1 affects the growth of intermediate and deep layer vasculature, we quantified vessel density in these layers. At P24.5, vessel density in the intermediate layer is reduced in Bmal1; CKO animals compared to controls (Fig. 1C-D′′ F, F′). Although the density is much lower in the deeper layer, it is not significantly different compared to controls (Fig. 1E-E′′, F, F′). Thus, loss of Bmal1 from the VECs results in an overall reduction in retinal vessel density.

### Deletion of endothelial period 2 (Per2) affects deep layer vasculature

**Fig. 2:**
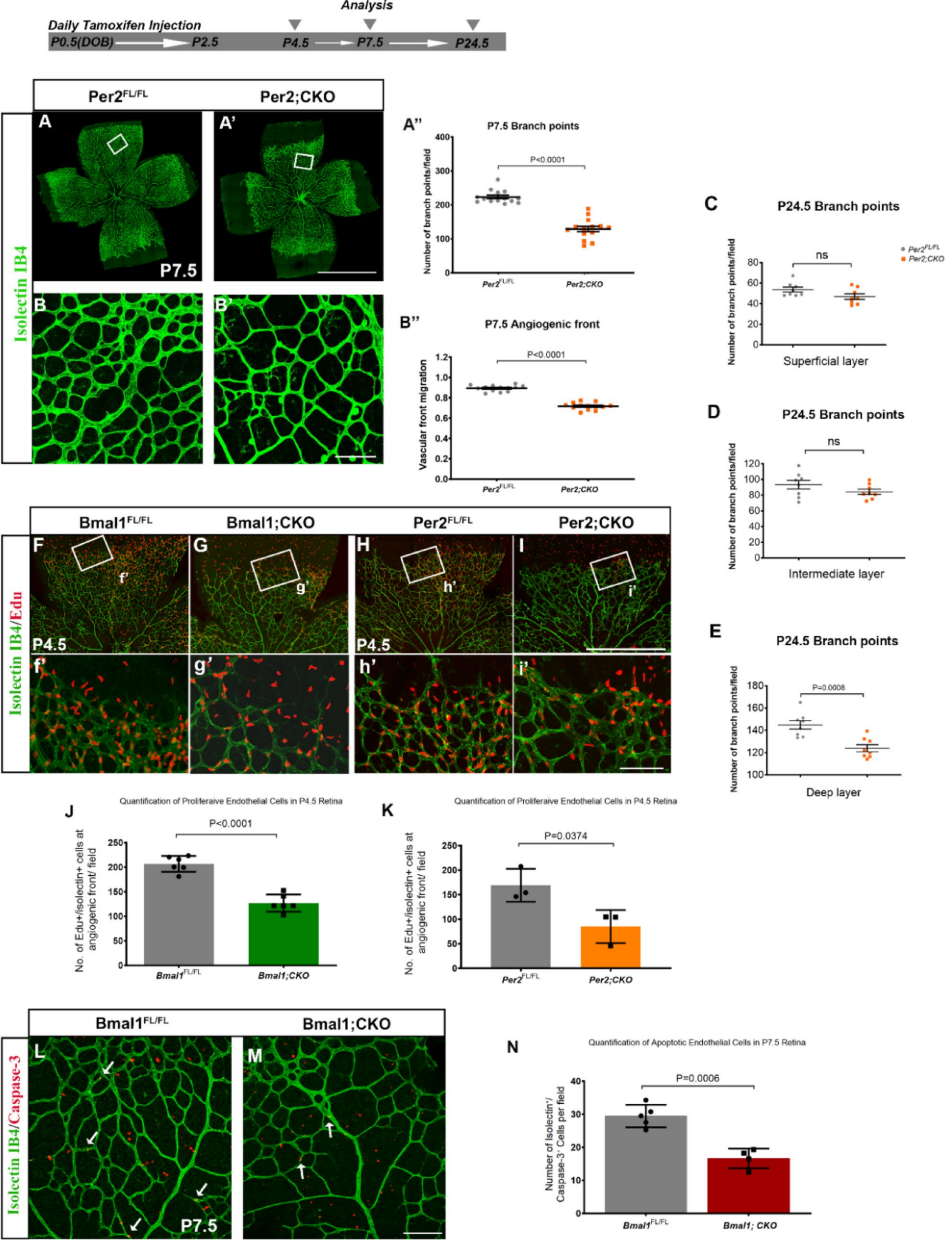
Per2 deletion transiently disrupts the superficial vascular layer and significantly affects the deeper layer: (**A**, **A**′) Retinal flat mounts stained with Isolectin IB4 to label endothelial cells. (**B**, **B**′) Representative higher magnification images of boxed regions to illustrate differences in vessel density at P7.5. (**A**′′, **B**′′) Per2;CKO animals show a significant decrease in angiogenic front migration and vessel density in the remodeling area when compared to Per2^FL/FL^. (**C**, **D**, **E**) Quantification of P24.5 vessel density in Per2^FL/FL^ vs Per2;CKO animals, from the superficial (C), intermediate (D), and deep (E) vascular layers. Vessel density in the superficial and intermediate layers is not significant, but the deeper layer is significantly reduced in the Per2;CKO animals. (**F**, **G**, **H**, **I**) Representative images from P4.5 retinal flat mounts stained with Isolectin-IB4 (green) to mark endothelial cells and EdU (red) as an S-phase proliferation marker. (**f**′, **g**′, **h**′, **i**′) Higher magnification images of the highlighted boxed regions reveal differences in EdU density between the groups. (**J**, **K**) Graph shows quantification of EdU + /Isolectin + cells from both Bmal1;CKO and Per2;CKO retinas, indicating a significant reduction in EdU + cells in both groups. (**L**, **M**) Field images of retinal wholemounts stained with Isolectin-IB4 (green) and Caspase-3 (red) from Bmal1^FL/FL^ control (L) and Bmal1;CKO mutant (M) retinas, white arrows indicate the Isolectin/caspase-3 + cells. (**N**) Mutant retinas show reduced numbers of Isolectin⁺/caspase-3⁺ (apoptotic endothelial) cells compared to controls. All retinas were collected at noon (ZT6). Significance was determined using a student’s t-test. Each data point represents a single eye. Error bars represent ± SEM. *n* = 3–14. The scale bars on flat mounts and field images are 800 µm (A′), 50 µm (B′, i′, M), and 100 µm (I), respectively

To determine whether Bmal1 regulates vascular development independent of its function as a component of the molecular clock, a similar strategy was used to delete Per2, the negative regulator of Bmal1/Clock heterodimer complex. Per2 conditional knockout (Per2; CKO) animals, at P7.5, phenocopy the vascular defects observed in the Bmal1; CKO animals with reduced vascular density (Fig. 2A, A′, A′′) and migration (Fig. 2B, B′, B′′). These effects are transient, and by P24.5, the superficial and intermediate layers of the Per2; CKO and the Per2^FL/FL^ retinas are similar (Fig. 2C, D) but the vessel density in the deeper layer is significantly lower (Fig. 2E). Deeper vascular layer develops before the intermediate layer; thus, reduced vessel density in the Per2; CKO retina is not due to a developmental delay but is more likely due to a specific role of Per2 in the growth of the deeper vascular layer. Reduced vessel density could be due to increased apoptosis, failure of cell proliferation, or both. To examine whether proliferation was affected, Bmal1; CKO and Per2; CKO animals were injected with EdU, an S-phase marker, at P4.5 or at P8.5/P9.5. As seen in Fig. 2F-i′, Bmal1; CKO (Fig. 2G, g′, J, Supplementary Fig. 5A-E) and Per2; CKO (Fig. 2I, i′, K, Supplementary Fig. 5F-J) animals show significantly lower numbers of S-phase positive cells compared to their respective controls (Fig. 2F, f′, H, h′, J, K), irrespective of the age when proliferation was assessed. Furthermore, there is a significantly lower number of caspase-3 positive apoptotic endothelial cells in Bmal1; CKO retinas compared to controls, thus eliminating cell death as a cause of reduced vessel density (Fig. 2L-N). Overall, this data suggests that circadian clock genes Bmal1 and Per2 regulate retinal VEC proliferation during developmental angiogenesis.

### Bmal1 regulates cell cycle-related transcriptional networks within the endothelial cells

**Fig. 3:**
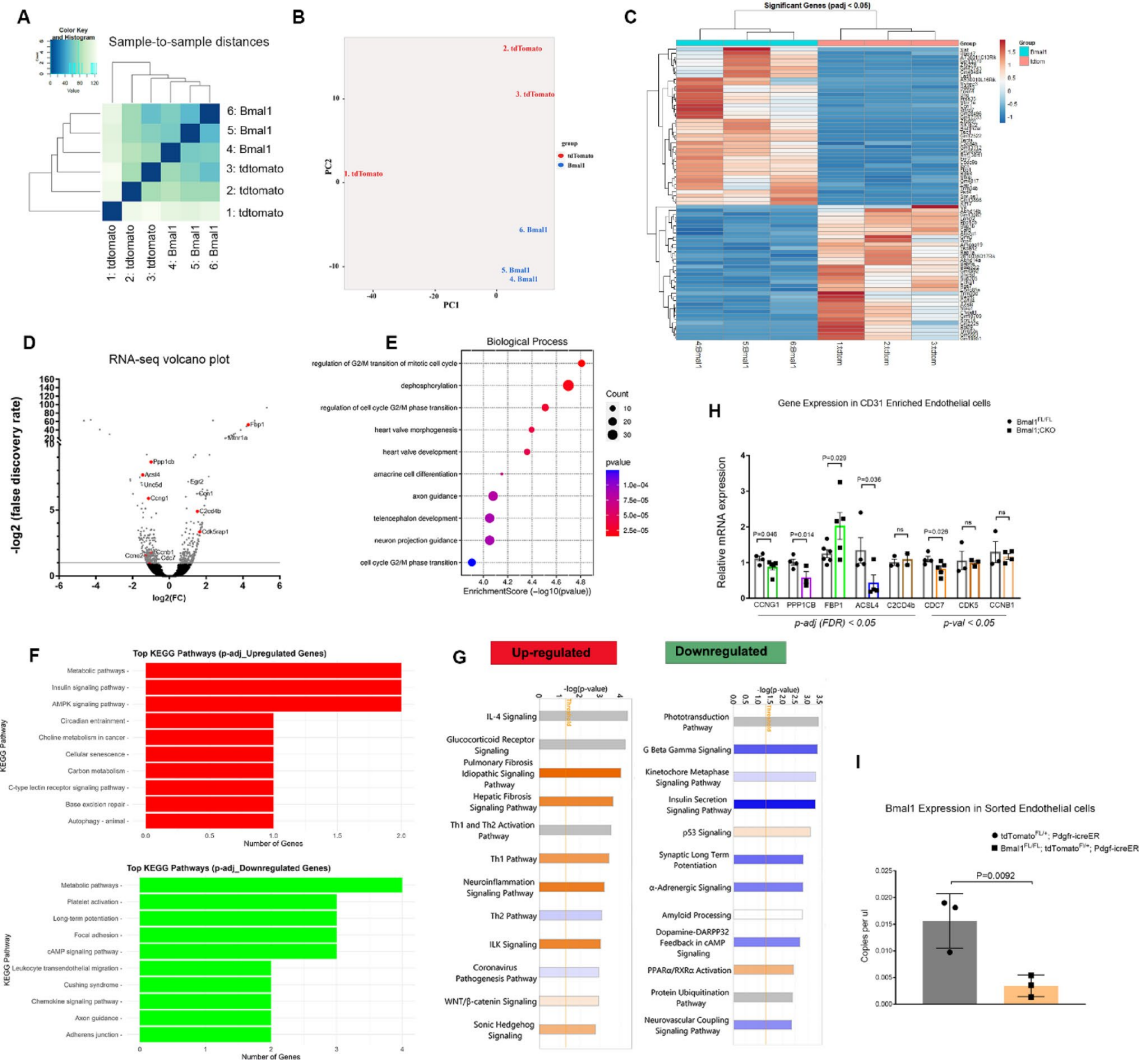
RNA-seq analysis and validation using q-RTPCR: (**A**, **B**) Pearson’s correlation (A) and Principal Component Analysis (B) were performed on normalized RNA-seq data to assess intra- and inter-group sample variability and identify any potential outliers. tdTomato represents the control group (tdTomato^FL/+^; Pdgf-icreER), and Bmal1 is mutant group (Bmal1^FL/FL^; tdTomato^FL/+^; Pdgf-icreER). (**C**) Heatmap represents differentially expressed genes (adjusted *p* < 0.05) between the two groups. Gene-wise Z-score normalization was applied to visualize relative expression changes across samples. The heatmap shows top 10 distinct gene expression profiles between control and Bmal1 mutant endothelial cells. (**D**) A volcano plot of differentially expressed genes, with a few genes labeled that are related to cell cycle, angiogenesis and circadian rhythms. (**E**) The gene ontology (GO) biological processes associated with Bmal1 targets are indicated. The x-axis represents the enrichment factor, and the y-axis represents the specific GO biological process. The bubble size indicates the number of targets enriched within each term, and color reflects the p-value. (**F**) Bar graph of top ten differentially expressed genes based on adjusted p-values, with upregulated genes shown in red and downregulated genes in green. (**G**) Ingenuity Pathway Analysis (IPA) represented as a horizontal bar graph illustrates the top upregulated and downregulated biological pathways based on changes in gene expression. (**H**) Quantitative RT-PCR data from CD31 bead-enriched endothelial cells show significant downregulation of differentially expressed genes associated with cell cycle regulation, like CDC7, CCNG1, PPP1CB, FBP1, and ACSL4. Other cell cycle genes like CCNB1, CDK5, and C2CD4b did not show any significant changes. β-actin was used as an internal reference control. (**I**) ddPCR quantification of Bmal1 copy numbers in the FAC-sorted enriched endothelial cells shows a significant reduction in Bmal1 transcript in the mutant (Bmal1^FL/FL^;tdTomato^FL/+^;Pdgf-icreER) compared to the control (tdTomato^FL/+^;Pdgf-icreER). *n* = 3–6. All retinas were collected at noon (ZT6). Error bars are represented by ± SEM. Two-tailed student’s t-test was used to determine significance, and each data point represents a single eye

To identify the endothelial-specific molecular pathways regulated by Bmal1, we performed transcriptomic analysis of isolated retinal VECs. Cre recombinase-mediated reporter labeling of endothelial cells was used as a strategy to FAC sort VECs from P7.5 retina, as previously reported [11]. Labeled endothelial cells represented approximately < 0.1% of the total retinal cells, which is an accurate reflection of the retinal endothelial cell population. Pearson correlation coefficient and unsupervised hierarchical clustering analyses (Fig. 3A) show a clear separation between control and Bmal1; CKO, which is further confirmed by principal-component analysis (Fig. 3B). Differential expression patterns between the groups are illustrated in the heatmap (Fig. 3C) with some genes highlighted in the volcano plot that are specific to cell cycle related pathways (Fig. 3D). For differential expression analysis, we considered genes with a false discovery rate (FDR) < 0.05 as significant, identifying 42 upregulated and 36 downregulated genes in Bmal1;CKO endothelial cells compared to controls that are listed in the heatmap and the volcano plot. These significant gene lists did not show any changes in the known clock gene components. This could be due to the stringent parameters used for the analysis. Thus, we also analyzed the data using a cutoff of *P* < 0.05. This analysis revealed 621 upregulated and 775 downregulated genes, which also included Period2, the negative regulator of Bmal1 (Supplementary Fig. 11).

Pathway enrichment analysis based on FDR < 0.05 revealed that the top 10 enriched pathways in Bmal1; CKO endothelial cells include metabolic pathways, circadian entrainment, and cellular senescence (Fig. 3F). When using a less stringent cutoff of *P* < 0.05, additional pathways such as Hedgehog signaling and thyroid hormone signaling, both of which are involved in circadian regulation were also enriched (Fig. 3G). Other prominently altered biological pathways include Wnt signaling, PPARα signaling, neurovascular coupling, p53 signaling, and cell cycle regulation (Fig. 3E, F, G). qPCR analysis of enriched endothelial cells further validated the transcriptional changes observed in the RNA-seq data. As shown in Fig. 3H, CCNG1, PPP1CB, FBP1, ACSL4, and C2CD4B were selected from the FDR-significant gene list, while CDC7, CDK5, and CCNB1 were chosen based on *P* < 0.05. Among these, CDC7, CCNG1, and PPP1CB, key regulators of the cell cycle, were significantly reduced in Bmal1; CKO endothelial cells. Additionally, ACSL4, involved in lipid metabolism, was downregulated, while FBP1, a gluconeogenic enzyme, was upregulated in Bmal1; CKO endothelial cells. Although CCNB1 is also reduced in Bmal1; CKO, the change is not statistically significant. Bmal1 expression levels are significantly reduced in sorted endothelial cells, confirming an effective deletion of the gene (Fig. 3I). To visualize retinal cells that express Bmal1 and to demonstrate loss of Bmal1 from endothelial cells, we co-labeled cryosections with a Bmal1 antibody and isolectin for the vasculature. The majority of Bmal1 immunolabeled cells were present in the ganglion cell layer (GCL) and inner nuclear layer (INL). However, it was difficult to determine whether those were endothelial cells, since very few endothelial cell nuclei could be detected in the sections. Overall, the Bmal1-labeled cells appear to be present both in the controls and the Bmal;CKO retinal sections, suggesting that Bmal1 expression in the rest of the neural retina was unaffected. (Supplementary Fig. 9).

### Endothelial Bmal1 regulates the diurnal rhythms of retinal cell proliferation

**Fig. 4:**
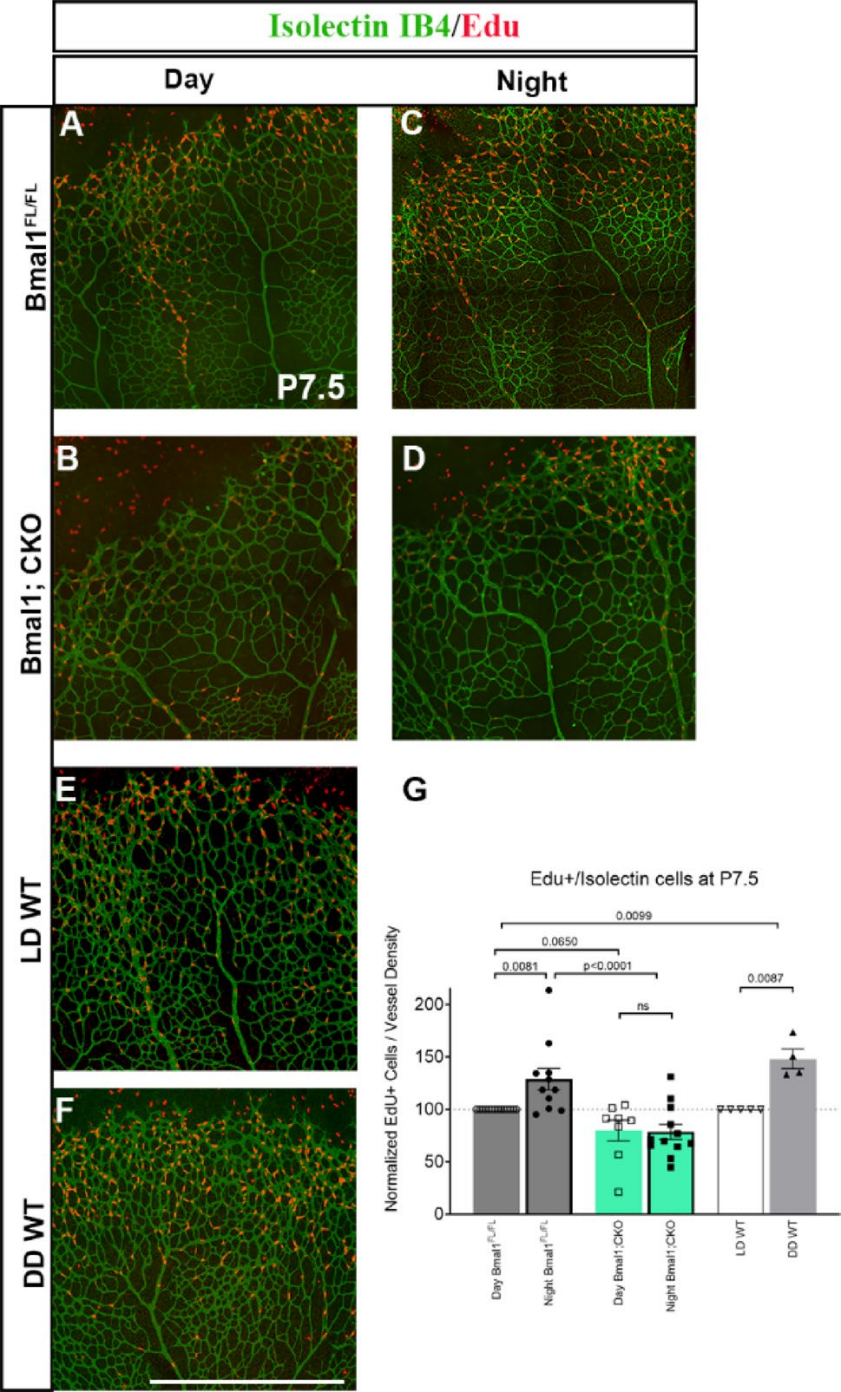
Vascular endothelial Bmal1 controls the diurnal regulation of retinal VEC proliferation: (**A**, **B**, **C**, **D**, **E**, **F**) Representative images of retinal wholemounts stained with Isolectin-IB4 (green) and EdU (red) from Bmal1^FL/FL^ animals during day (ZT6, A) and night (ZT18, C), Bmal1;CKO during day (B), night (D), wild-type animals maintained under a standard light/dark (LD, 14:10 h) cycle (E), and wildtype animals raised in the dark till P7.5 (F). (**G**) Graph represents the quantification of EdU + /isolectin cells from animals of the indicated genotypes. EdU + cell counts were normalized to vessel density, with the day Bmal1^FL/FL^ and LD WT set to 100%. Bmal1.^FL/FL^ animals demonstrate diurnal variations in proliferation, with a higher number of EdU + cells at night (C, G) compared to the day (A, G). Bmal1; CKO animals do not show any significant difference in the numbers of the EdU + cells between day (B, G) and night (D, G). (G) Compared to animals maintained under a standard cycle, animals raised in constant darkness (DD) showed a significantly higher number of EdU + cells in the retina, similar to control animals analyzed during the dark phase (C, G). Statistical significance was determined using two-way ANOVA using Fisher LSD and each data point represents a single eye. Scale bar: 100 µm. Error bars represent ± SEM, *n* = 4–13

Given the strong correlation between the cell cycle and circadian clock, we investigated whether retinal VEC proliferation is differentially regulated between the day and the night. We labeled cycling VECs during the day (at ZT6, where ZT0 represents light onset) and at night (ZT18) and quantified the number of proliferating endothelial cells. At ZT6, in control retinas, most of the EdU positive (EdU +) cells were concentrated in the peripheral retina, with very few EdU + cells detected in the central region. Conversely, at night, there is an overall higher number of EdU + cells, with many of them localized in the central retina (Fig. 4A, C, G). To account for potential variations in EdU + cells due to differences in vascular density, we normalized EdU + cell counts to vessel density. This analysis revealed a robust diurnal regulation of vascular endothelial cell proliferation, with a peak occurring at night. In contrast, VEC proliferation was reduced overall in the Bmal1; CKO retina, regardless of the time of the day (Fig. 4B, D, G). To determine whether this proliferative rhythm in the control retinas was dependent on light cues or driven by endogenous circadian mechanisms, we examined EdU incorporation in pups raised under constant darkness. In the absence of external light, there is a higher number of EdU + cells in the retina compared to controls that were raised under normal lighting conditions, indicating that retinal endothelial proliferation is regulated by a light-dependent mechanism (Fig. 4E, F, G). Overall, this data suggests that retinal endothelial cell proliferation in mice is differentially regulated between light–dark cycles, with more cells proliferating during the dark cycle.

### Bmal1 in retinal endothelial cells is required to regulate RGC number

**Fig. 5:**
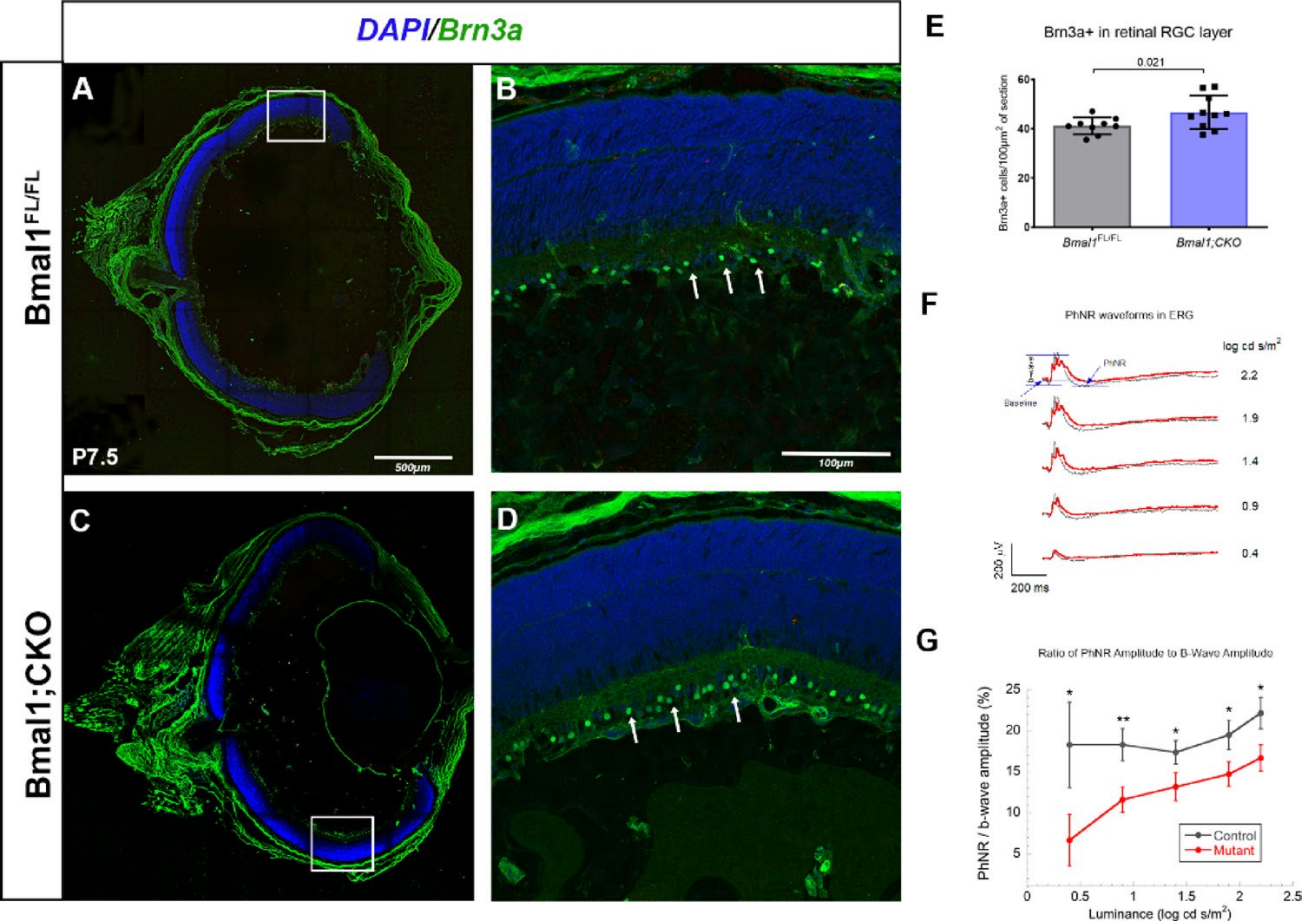
Loss of endothelial Bmal1 results in an increase in Brn3a positive retinal ganglion cell numbers accompanied by functional deficits: (**A**, **C**) Retinal frozen sections from Bmal1^FL/FL^ (A) and Bmal1;CKO animals (C), at P7.5 labeled with RGC marker Brn3a (Green) and nuclear stain DAPI (Blue). (**B**, **D**) Higher magnification images of the boxed area, with white arrowheads indicating the Brn3a + cell, which are higher in numbers in Bmal1; CKO retinas (**D**, **E**) compared to controls (B, E). (**F**) A representative PhNR (photopic negative response) waveform of the electroretinogram (ERG) recorded from a Bmal1^FL/FL^ (grey) and Bmal1; CKO (red) at different luminance intensities. (**G**) The ratio of the PhNR amplitude to the b-wave amplitude was calculated to assess RGC function at different luminance intensities. Error bars represent ± SEM, *n* = 4–10. Statistical analysis for Brn3a counts was done using Student’s t-test and each data point represents a single eye. For the PhNR analysis, a two-way repeated measures ANOVA was conducted to assess differences in the PhNR to b-wave amplitude ratio between mutant and control groups across five stimulus luminances

The changes in vessel density in the superficial layer of Bmal1; CKO retina raised the possibility that other cells, like RGCs and astrocytes, which are close to the superficial layer, could be indirectly affected. We labeled P7.5 retinal frozen sections with Brn3a, a pan retinal ganglion cell marker, and glial fibrillary acidic protein (GFAP) to mark astrocytes. It was difficult to determine whether the GFAP-labeled cells in Bmal1;CKO mutants were different due to the sparse labeling of the astrocyte processes in the retinal sections (Supplementary Fig. 2); however, the number of Brn3a⁺ RGCs was significantly increased compared to controls (Fig. 5A–E). To investigate whether these changes persist in adults, Brn3a + RGCs were also counted at P24.5. Although the numbers remain elevated, the difference is not statistically significant (Supplementary Fig. 8). In contrast, Per2; CKO animals show no difference in Brn3a⁺ RGC numbers compared to controls at P24.5 (Supplementary Fig. 10). This data suggests that loss of Bmal1 in retinal endothelial cells can have an impact on the RGC numbers postnatally.

### Loss of Bmal1 impairs RGC function as measured by photopic negative response (PhNR)

To assess whether the increased number of Brn3a + cells in Bmal1;CKO retinas translates into functional deficits, we recorded photopic visual responses using full-field electroretinography (ERG). The scotopic a-wave/b-wave and photopic b-wave responses were unaffected in Bmal1; CKO animals (Supplementary Fig. 3), which is expected since these ERG components primarily reflect photoreceptor and bipolar cell activity, respectively, rather than RGC function. In contrast, the PhNR, a negative ERG component generated by RGCs, showed reduced amplitudes in Bmal1; CKO animals, although the reduction of PhNR amplitude did not reach statistical significance compared with controls. Notably, the ratio of PhNR to b-wave amplitude was significantly decreased in Bmal1; CKO animals (Fig. 5F, G). These findings give credence to the idea that the circadian clock within the retinal vasculature modulates RGC function by regulating both their number and responsiveness.

## Discussion

Taken together, this data strongly supports the role for the circadian clock genes Bmal1 and Per2 in regulating retinal angiogenesis. Importantly, Bmal1 and Per2 exert a layer specific effect on angiogenesis, with Bmal1 predominantly regulating superficial and intermediate layer angiogenesis, while Per2 influences the development of the deeper layer. These layer specific effects may be due to bidirectional communication between the vasculature and the underlying neuronal cells. Our data also demonstrates that vascular endothelial cell proliferation is dependent on the light/dark cycle. RNA-seq data from enriched retinal endothelial cells show that Bmal1 regulates VEC cell cycle during angiogenesis. Furthermore, endothelial Bmal1 has a non-cell autonomous function in regulating RGC numbers and thus function.

*Bmal1 and Per2 in the vasculature*: Endothelial cell-specific deletion of Bmal1 and Per2 has similar effects on retinal angiogenesis, with the loss of either leading to stunted growth and reduced endothelial cell proliferation. Although Bmal1 and Per2 function as positive and negative regulators of the circadian clock, respectively, the deletion of any circadian clock components can produce comparable outcomes. Notably, Per2 knockout (KO) retinas exhibit reduced Bmal1 expression, suggesting that vascular defects observed in Per2; CKO are partially mediated by the suppression of Bmal1 (Supplementary Fig. 7). This may explain the similar vascular phenotypes observed in both Bmal1; CKO and Per2; CKO retinas. It is important to highlight that despite certain similarities in the effects of Bmal1 and Per2 on retinal vascular growth, there are distinct differences between these two circadian clock regulators. For example, in the Per2; CKO, retina, the superficial layer recovers by P24.5, but not the deeper layer vasculature. These effects on deeper layer vasculature cannot be due to a delay in vascular development, as the deeper layer vessels develop before the intermediate layer and the intermediate layer remains unaffected in the Per2;CKO animals. Thus, circadian clock components can have layer-specific effects on retinal vascular growth. The extent to which these clock components regulate the function of these vascular layers requires further investigation.

This analysis also reveals species-specific differences in the role of the circadian clock in vascular development. For example, in zebrafish, loss of Bmal1 and Per2 results in opposing effects on vascular growth [28], whereas in this study, loss of Bmal1 and Per2 in endothelial cells has similar effects on physiological angiogenesis. Our findings are also supported by recent work, which demonstrated that endothelial cells possess a functional circadian clock and that BMAL1 regulates EC proliferation and angiogenesis through direct transcriptional control of cell cycle genes such as CCNA1 and CDK1. This study complements our observations and supports the role of endothelial circadian regulation as a critical determinant of vascular development and function [14]. In agreement with our data, other studies have reported similar effects on vasculature with loss of Per2. For example, endothelial cells from Per2^m/m^ mice show impaired vascular network formation and cell proliferation [29]. In these Per2 mutants, the deletion of the PAS-B domain results in a shorter circadian period, followed by complete loss of circadian rhythmicity in constant darkness [7]. In another study, endothelial progenitor cells (EPC) from Per2^−/−^ mice demonstrate a significant reduction in their ability to migrate in response to pro-migratory signals compared to wild-type EPCs [30]. Thus, regardless of the species, the circadian clock genes play a critical role in regulating vascular development.

*Bmal1 and Cell Cycle*: We have previously reported that Bmal1 regulates the timing of retinal neuronal cell cycle entry and exit [31]. In this study, we show that Bmal1 also regulates retinal endothelial cell cycle. Interestingly, irrespective of the cellular identity, one core function of the circadian clock gene is to regulate cell proliferation and thus control the temporal wave of cellular specification. This intricate relationship between circadian clocks and cell cycle dynamics underscores the significance of such interaction in maintaining cellular homeostasis and influencing disease outcomes. Our RNA-seq data indicate that Bmal1 modulates the expression of key cell cycle regulators such as cyclins and cyclin-dependent kinases (CDKs), thereby influencing the timing of cell cycle transitions. In line with these findings, the most significantly altered biological pathways in Bmal1;CKO endothelial cells include Wnt signaling, PPARα signaling, Insulin signaling, thyroid hormonal signaling, neurovascular coupling, p53 signaling, and cell cycle regulation. These pathways are known to play important roles in angiogenesis, endothelial proliferation, metabolic regulation, and vascular stability. Their disruption likely contributes to the reduced vessel density observed in Bmal1; CKO retinas, highlighting the central role of Bmal1 in coordinating multiple signaling cascades required for proper retinal vascular development. Although this effect is observed during development, circadian clocks exert similar effects on the cell cycle in adults. Studies have demonstrated that circadian disruption can alter the expression of tumor suppressor genes and oncogenes, thereby influencing cancer progression. Misalignment between the circadian clock and cell cycle could lead to aberrant cell proliferation, increased susceptibility to genomic instability, and a higher risk of cancer [32, 33]. Aberrant angiogenesis is also a hallmark of several retinal pathologies [34]. It is conceivable that a disrupted endothelial clock contributes to the abnormal proliferation in these retinal diseases. The retina serves as a good model system to investigate the relationship between the cell cycle and the circadian clock.

One notable effect we observed in the retina was the synchronization of cell proliferation with the daily light/dark cycle. Similar temporal regulation of cell proliferation has been reported for the interfollicular epidermal (IFE) stem cells, which in mice, peak at night (ZT17-21) and is minimal during the day (ZT5-9) [35]. Why cellular proliferation is diurnally regulated remains to be an area of active investigation. Bmal1 has an impact on the cell cycle, affecting diurnal cell proliferation and overall cellular function. Understanding these interactions will provide valuable insights into the mechanisms underlying circadian rhythm-related diseases. This offers potential avenues for therapeutic interventions aimed at restoring circadian alignment and improving cellular health.

*Neurovascular coupling and refinement of retinal cell numbers*: A common feature of the central nervous system (CNS) is the overproduction of neuronal cells, which are then refined based on cellular and sensory signals. The retina is no exception; like in the CNS, retinal neuronal cells are produced in higher numbers and undergo sensory input-based refinements [36]. Similarly to the CNS, many neuronal cells are eliminated by apoptosis. RGCs are the earliest born neurons and experience a significant decrease in density postnatally. This reduction is important for RGC function [37–39]. Therefore, if an RGC axon fails to innervate its target properly, it is eliminated due to deprivation of trophic factors necessary for survival. This refinement process depends on neurotrophic factors released from synaptic terminals. However, there is evidence suggesting these neurotrophic factors can also be intraretinal, and RGCs might themselves produce these factors. The neurotrophic factors involved in developmental apoptosis include the neurotrophin family, which comprises six related factors: Brain-Derived Neurotrophic Factor (BDNF), Nerve Growth Factor (NGF), Neurotrophin-3, Neurotrophin–4/5, Neurotrophin-6, and Neurotrophin-7. These factors bind to two families of transmembrane receptors: the TRK receptors within the tyrosine kinase family and the extracellular domain of the p75 neurotrophin receptor [40]. Both TrkB and BDNF are expressed by non-neuronal tissues such as the heart, fat, and vasculature, at levels comparable to those in the brain. Loss of BDNF also results in vascular defects [41–45]. In this analysis, loss of Bmal1 in endothelial cells is linked to altered RGC numbers, possibly due to impaired proliferation or reduced apoptosis. Further research is needed to understand the underlying mechanisms. However, our data suggest that loss of Bmal1 in vascular endothelial cells plays a key role in regulating neuronal numbers and functions via paracrine signaling [46]. How this effect is mediated remains unknown, but it could be a focus for future investigation.

## Supplementary Information

Below is the link to the electronic supplementary material.

Supplementary Material 1

Supplementary Material 2

Supplementary Material 3

Supplementary Material 4

## Data Availability

The authors declare that data supporting the findings of this study are available within the article, and its supplementary information files. Raw data and related files for RNA-seq are deposited in GEO, and the accession number is GSE284799.
