# Supplementary material for: Endothelial clock regulates retinal angiogenesis and ganglion cell function
Source: Angiogenesis. 2025 Nov 15;29(1):6. doi: 10.1007/s10456-025-10018-4 (PMC12619741; doi:10.1007/s10456-025-10018-4)
Supplement: Supplementary file 2 — Supplementary Material 2 [file 10456_2025_10018_MOESM2_ESM.pdf]

**Supplementary Figure 1:**

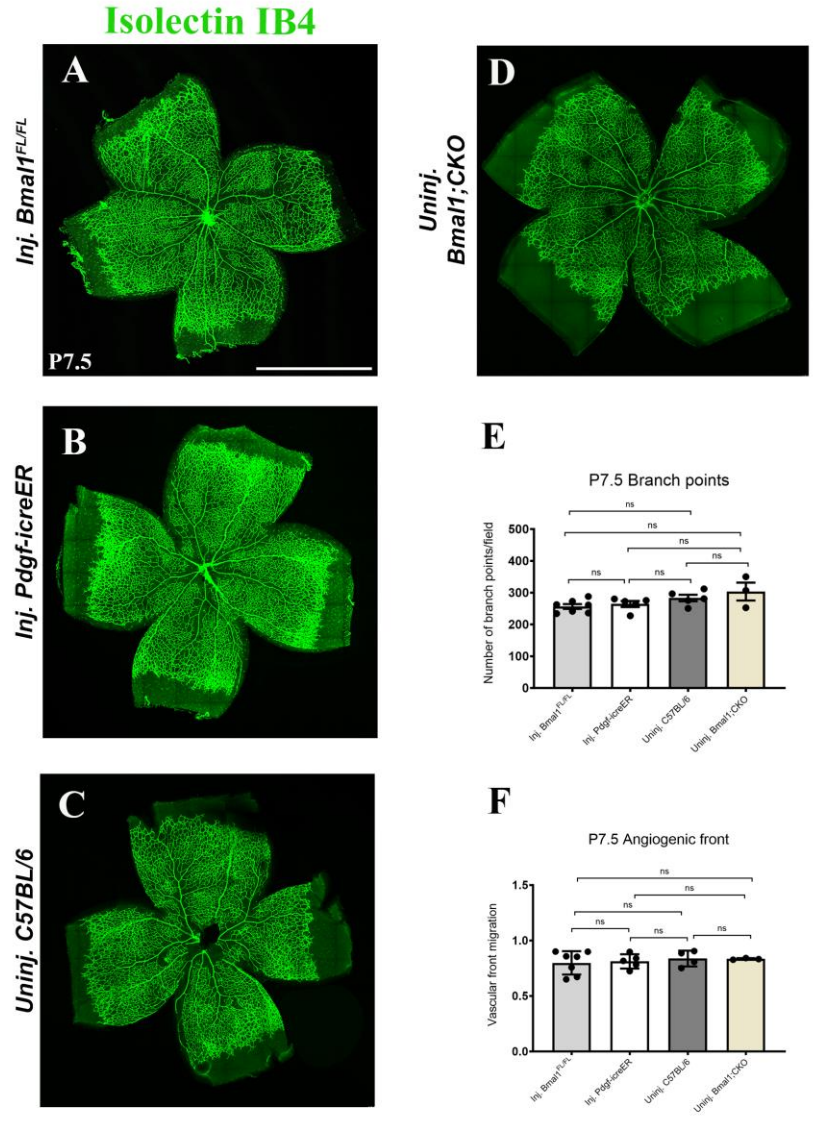


**Supplementary Fig. 1: Validation for Cre basal expression without induction and non-specific effects of tamoxifen injection on retinal angiogenesis:**

(A, B) Images of the retinal whole mounts labeled with Isolectin from Bmal1$^{FL/FL}$ and Pdgf-icreER animals at P7.5. Animals were injected with tamoxifen for three consecutive days starting on the day of birth (P0.5). (C, D) Uninjected C57BL/6 and Bmal1;CKO animals were used to assess baseline vascular development and potential Cre expression without induction. (E, F) A comparison of vessel density and angiogenic front migration between both tamoxifen-injected and uninjected groups revealed no significant differences, indicating that the Pdgf-icreER transgene and tamoxifen treatment do not independently affect retinal vascular development in the absence of Bmal1 deletion. Error bars represent ±SEM. n=3-7. The tissue was collected at ZT6. Statistical significance was calculated using one-way ANOVA. Scalebar=800µm.

**Supplementary Figure 2:**

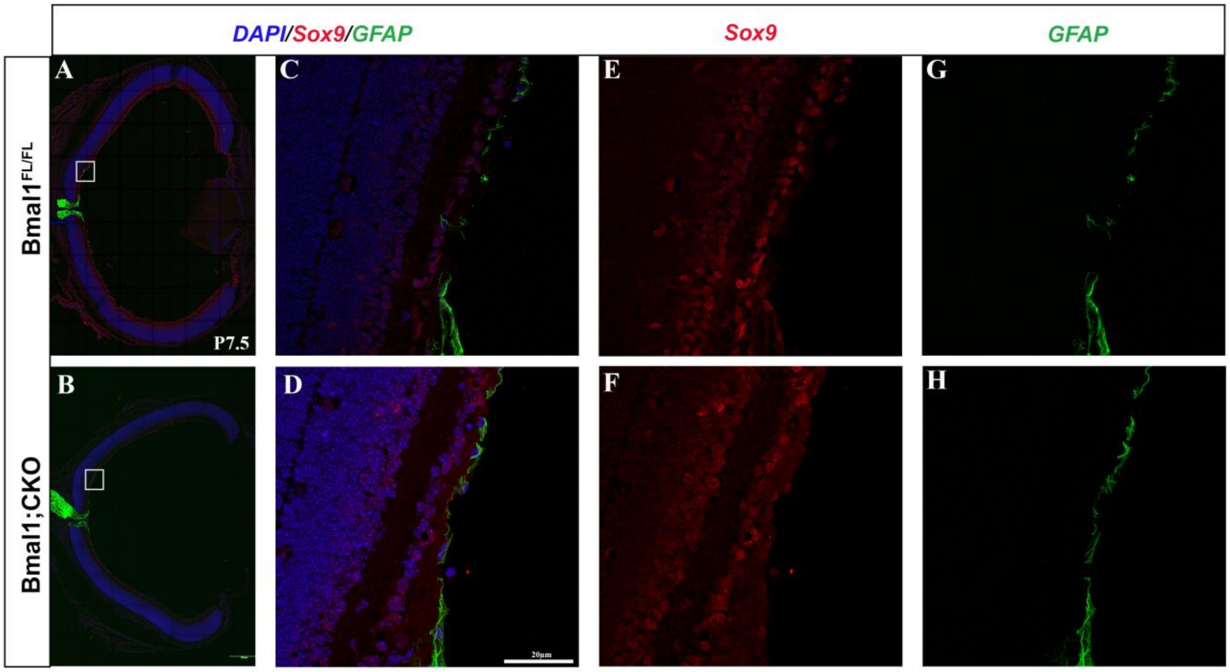


**Supplementary Fig. 2: No observable changes in Sox9 and GFAP staining in retinal sections from Bmal1; CKO and Bmal1$^{FL/FL}$ animals.**

(A, B) Representative retinal sections at P7.5 labeled with DAPI (blue), Sox9 (red, astrocyte nuclei), and GFAP (green) for astrocytes from Bmal1$^{FL/FL}$ (control) and Bmal1$^{FL/FL}$; CKO (mutants) groups. Staining patterns appear to be similar between the two groups (C-H). n=3. Scale bar: 75 µm (B), 20 µm (D).

**Supplementary Figure 3:**

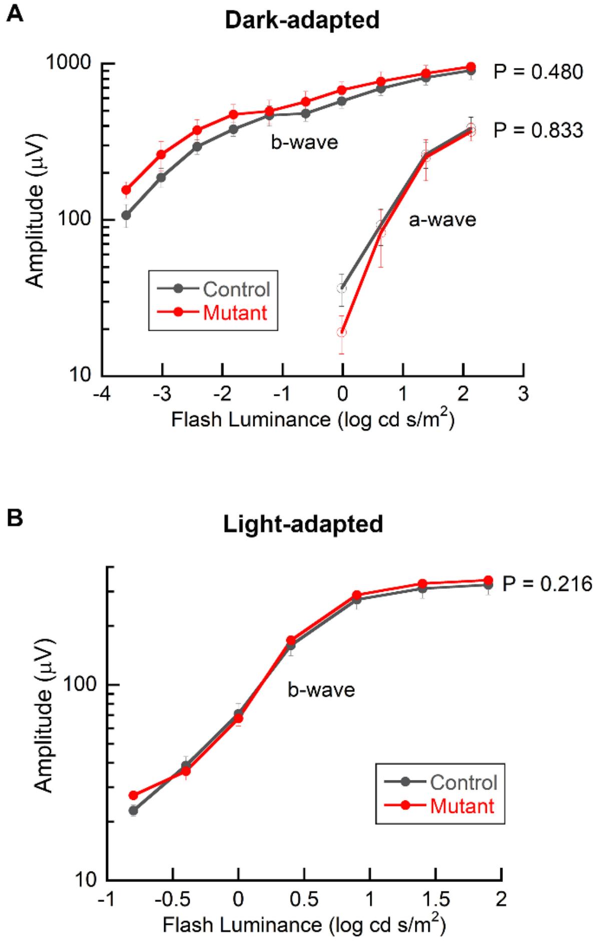


**Supplementary Fig. 3: Electroretinographic a- and b-wave amplitudes in control Bmal1$^{FL/FL}$ mice and Bmal1:CKO mice at P30.**

(A, B) Graphs representing the amplitudes of dark-adapted a- and b- waves at different luminance intensities recorded from Bmal1$^{FL/FL}$ (control) and Bmal1$^{FL/FL}$;CKO (mutants) groups at P30. (A) Dark-adapted ERG recordings show that b-wave amplitudes, generated primarily by rod ON-bipolar cells, and a-wave amplitudes, originating from rod photoreceptors, did not differ significantly between groups across a range of flash luminances. (B) Light-adapted ERG b-wave amplitudes, reflecting cone pathway activity, also showed no significant differences between groups. Data are presented as mean ± SEM; n = 4–6 per group. Statistical comparisons were performed using repeated-measures ANOVA with post-hoc Bonferroni tests.

**Supplementary Figure 4:**

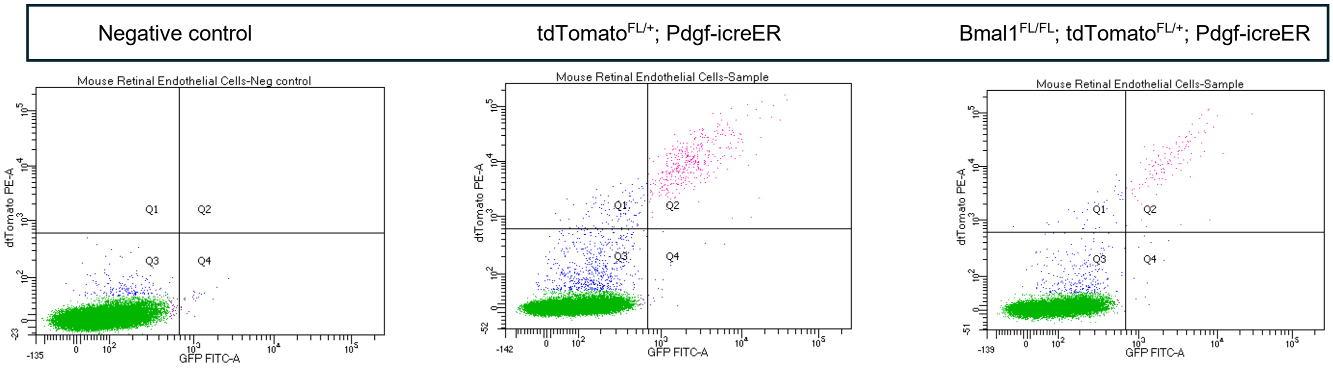


**Supplementary Fig. 4: FACS analysis of endothelial cells using GFP and tdTomato markers**

Flow cytometry analysis of endothelial cells sorted based on double-positive expression of GFP (driven by Pdgf-icreER) and tdTomato reporter. The negative control sample does not have any double-positive cells, confirming the specificity of the gating strategy. Both the control (tdTomato$^{FL/+}$; Pdgf-icreER) and mutant (Bmal1$^{FL/FL}$; tdTomato$^{FL/+;}$ Pdgf-icreER) samples contain clear populations of GFP+/tdTomato+ double-positive cells, indicating successful labeling of endothelial cells in both groups.

**Supplementary Figure 5:**

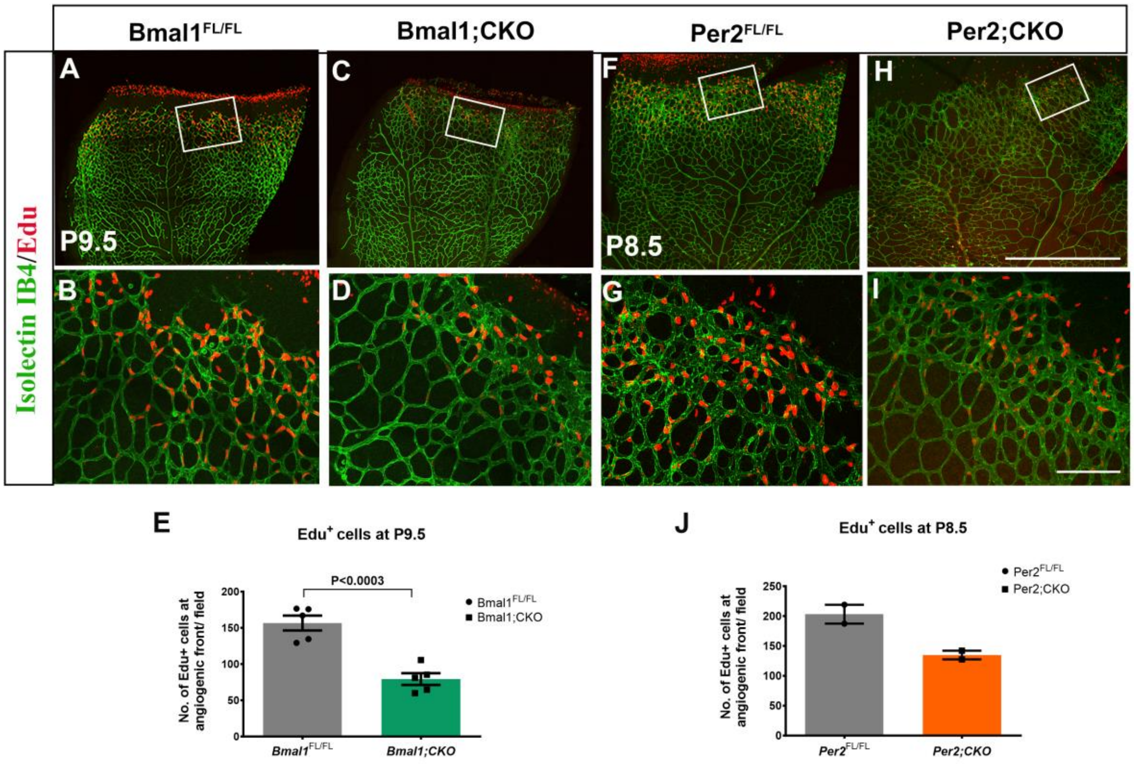


**Supplementary Fig. 5: Reduced endothelial cell proliferation in Bmal1;CKO and Per2;CKO retinas**

(A, C, F, H) Representative images of retinal flat mounts from P8.5 and P9.5 animals labeled with Isolectin-IB4 (green) to visualize endothelial cells and EdU (red) to mark S-phase proliferating cells. (B, D, G, I) Higher magnification images of the boxed regions highlight differences in the number of EdU-positive cells between groups. (E, J) Quantification reveals a significant reduction in EdU-positive cells in Bmal1;CKO compared to the controls. Although the sample size is very low for Per2;CKO and the control retinas, in general, there is a reduction in EdU+ cells in the Per2;CKO animals as well. Statistical significance was determined using Student's t-test. Error bars represent ± SEM; n = 2–5.

**Supplementary Figure 6:**

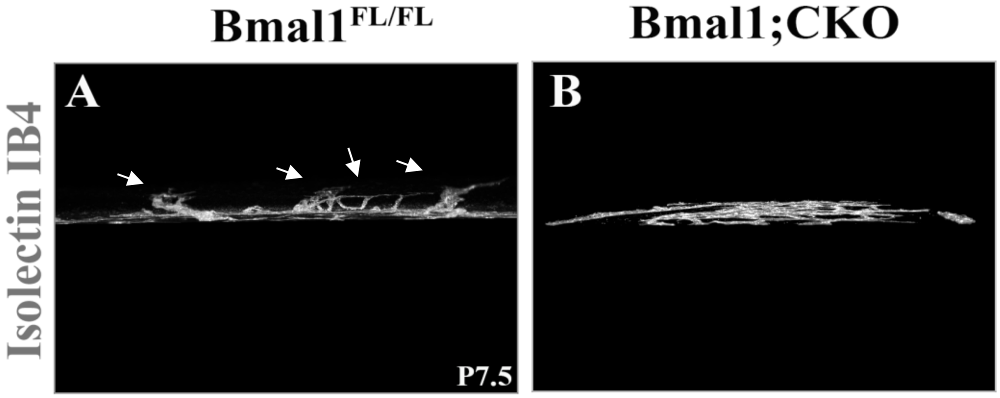


**Supplementary Figure 6. Delayed vascular sprouting in Bmal1; CKO retinas at P7.5**

Retinal field images of the superficial retinal vasculature at P7.5 stained with Isolectin-IB4 to visualize endothelial cells. (A) Bmal1$^{FL/FL}$ control retinas show several vertical sprouts (white arrows) from the superficial layer, indicating ongoing vascular development. (B) Bmal1;CKO retinas display reduced sprouting, suggesting delayed or impaired angiogenic progression in the absence of Bmal1. Images represent 3D projections of the superficial vascular layer.

**Supplementary Figure 7:**

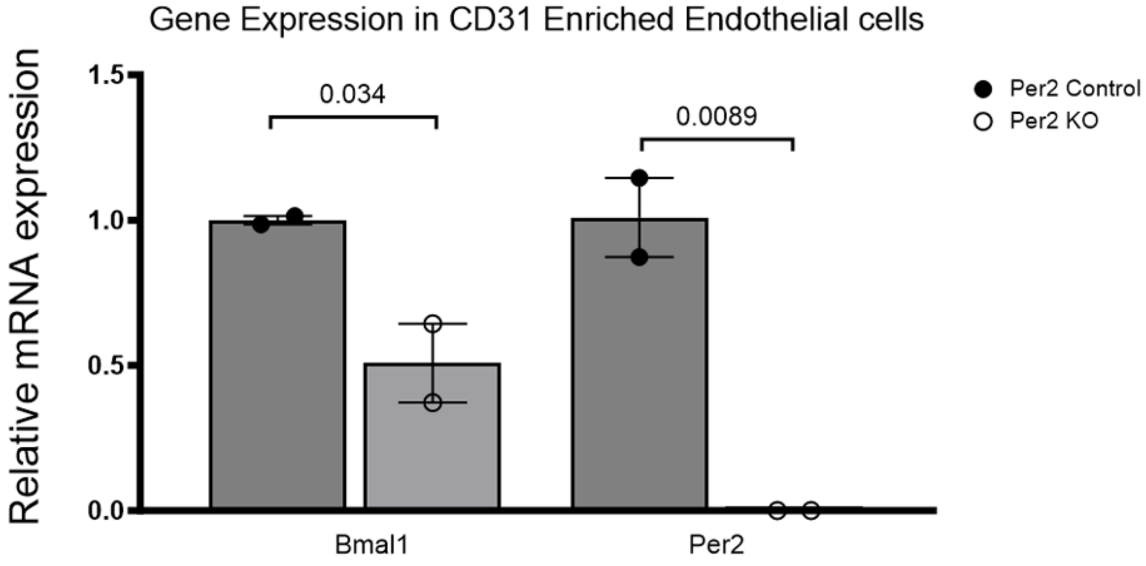


**Supplementary Figure 7. qPCR analysis of Bmal1 and Per2 expression in CD31-enriched endothelial cells from Per2 control and knockout retinas**

Bmal1 expression was significantly decreased in Per2 KO enriched endothelial cells compared to Per2 controls. Per2 expression was abolished in Per2 KO cells, confirming successful gene deletion. Data are presented as mean ± SEM. Statistical significance was determined using student's t-test. n=2

**Supplementary Figure 8:**

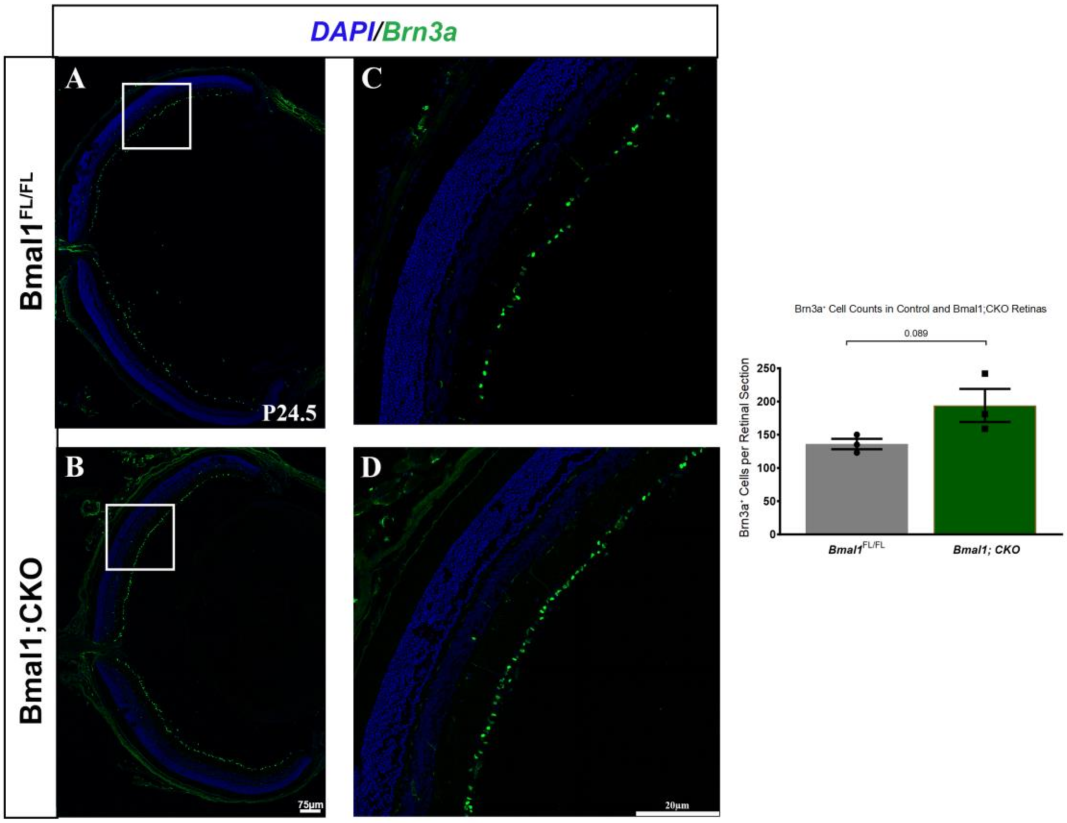


**Supplementary Figure 8. Brn3a⁺ cells in Bmal1; CKO retina remain elevated at P24.5**

(A, B) Representative images of whole retinal cryosections stained with DAPI (blue, nuclei), and Brn3a (green, retinal ganglion cell marker) from Bmal1$^{FL/FL}$ control (A) and Bmal1; CKO mutant (B) mice. (C, D) Higher magnification field images of the boxed regions in (A) and (B), respectively, indicate the ganglion cell layer. Overall, there are more Brn3a$^{+}$ cells in the Bmal1; CKO retina (D) compared to the control (C), suggesting there is an effect on the retinal ganglion cell population in mutants at P24.5. Data are presented as mean ± SEM. Statistical significance was determined using a student t-test. Scale bars: 75 µm (B) and 20 µm (D). n=3.

**Supplementary Figure 9:**

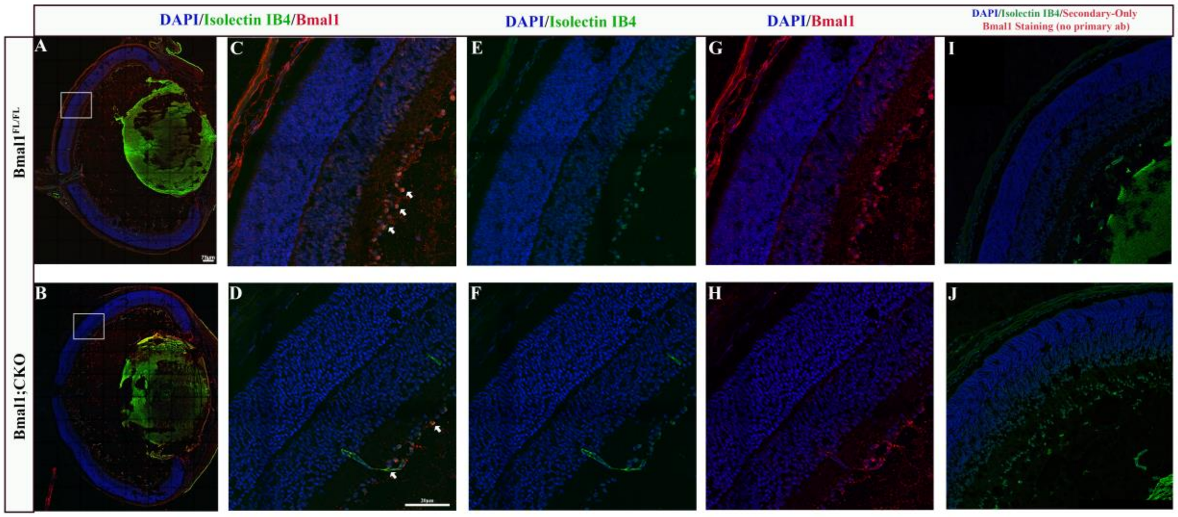


**Supplementary Figure 9. Bmal1 immunostaining in control and endothelial-specific Bmal1 knockout retina**

(A, B) Whole retinal cryosections from Bmal1$^{FL/FL}$ control and Bmal1;CKO mutant mice stained with DAPI (blue, nuclei), Isolectin (green, endothelial marker), and Bmal1 (red). (C, D, E, F, G, H) Higher magnification of the boxed regions in (A) and (B). Bmal1 immunoreactivity is detectable in the ganglion cell layer (GCL) and inner nuclear layer (INL), however, clear colocalization of Bmal1 with endothelial cells is difficult to determine. A few potentially colabeled cells are indicated with white arrowheads. (I, J) Secondary antibody-only control (no primary antibody) and mutant shows no background signal under identical imaging settings, validating the specificity of the Bmal1 staining. Scale bars: 75 µm (A), 20 µm (D).

**Supplementary Figure 10:**

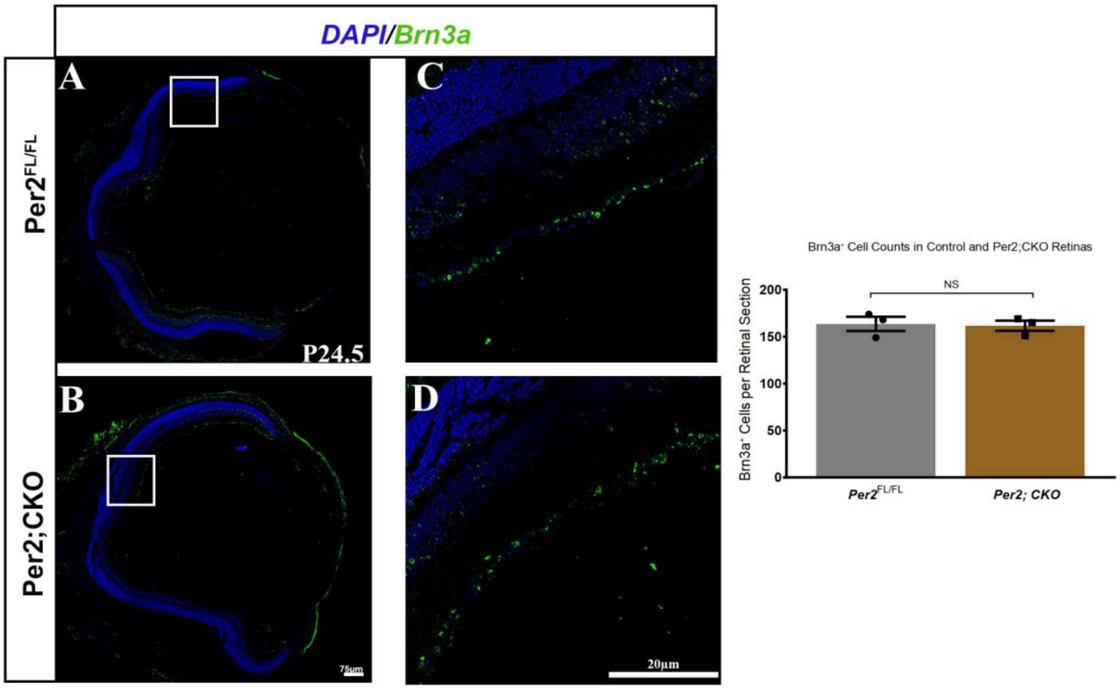


**Supplementary Figure 10. Brn3a⁺ Retinal ganglion cells are unchanged in Per2;CKO retinas compared to controls**

(A, B) Whole retinal cryosections from Per2$^{FL/FL}$ control (A) and Per2;CKO mutant (B) mice stained with DAPI (blue, nuclei) and Brn3a (green, retinal ganglion cell marker). (C, D) Higher magnification images of the boxed regions in (A) and (B), respectively, showing the ganglion cell layer. No noticeable differences in the number of Brn3a$^{+}$ cells were observed between control and Per2;CKO retinas. Statistical significance was determined using Student's t-test. Error bars represent ± SEM; Scale bars: 75 µm (B) and 20 µm (D). n = 3.

**Supplementary Figure 11:**

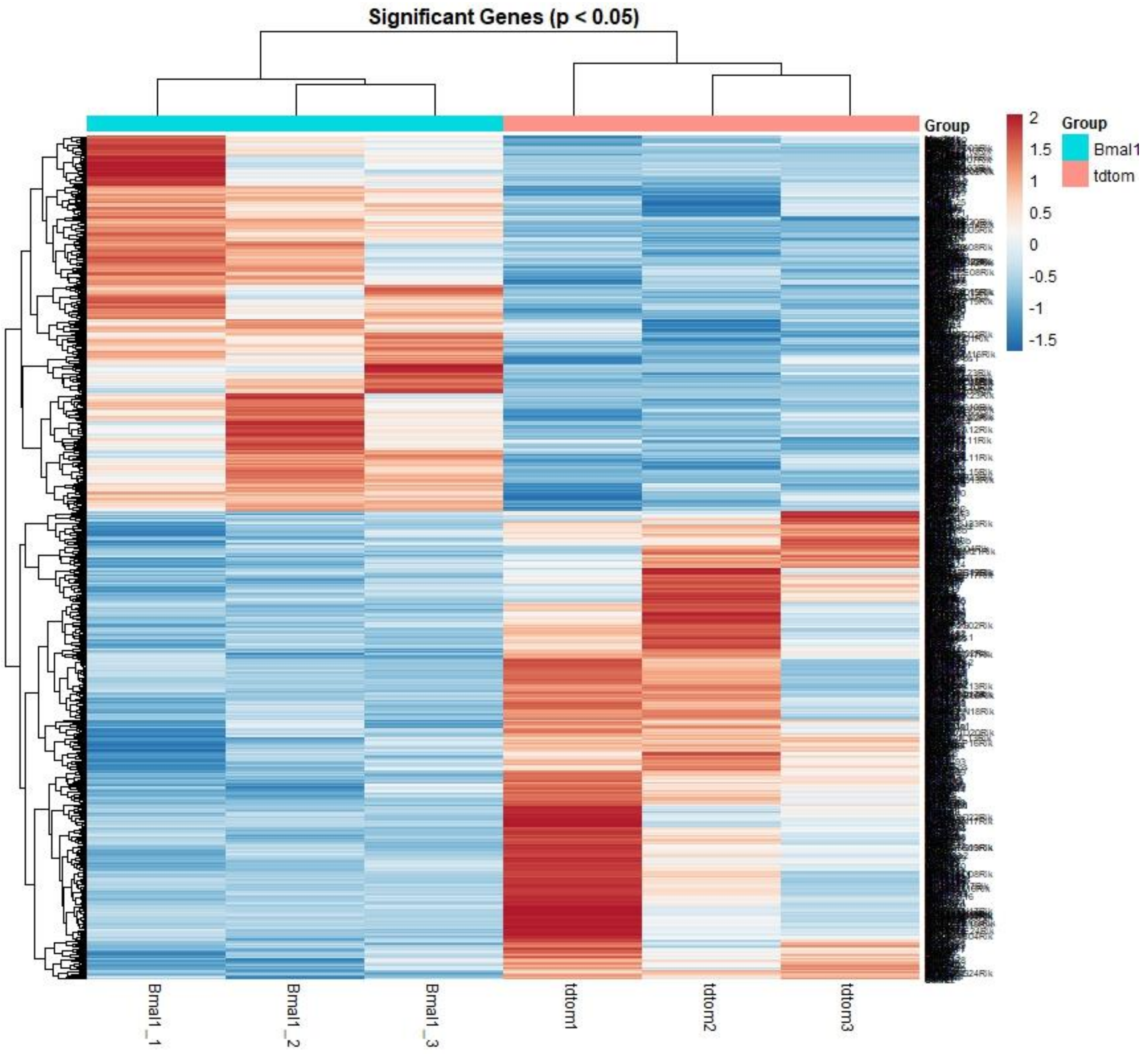


**Supplementary Figure 11: DEG Heatmap (p < 0.05, Gene-wise Z-score)**

Heatmap represents differentially expressed genes p-value < 0.05 between the two groups. Gene-wise Z-score normalization was applied to visualize relative expression changes across samples.

**Supplementary Table 1:**

| Gene | Forward Primer | Reverse Primer |
|---|---|---|
| Bmal1 | 5'-GGCCCACAGTCAGATTGAAA-3' | 5'-GCTGAACAGCCATCCTTAGC-3' |
| β-actin | 5'-TTCTTTGCAGCTCCTTCGTT-3' | 5'-ATGGAGGGGAATACAGCCC-3' |
| CDC7 | 5'-ACCGTGATGTGAAGCCCAGCAA-3' | 5'-CAGACTGGACAAACTTGAGCAGC-3' |
| CCNG1 | 5'- AAGACGTGGCTGTCAAGATGA-3' | 5'-GCCATTATCATGGGCCGACT-3' |
| PPP1CB | 5'- CTCGTGAAATCTTTCTCAGCCAG-3' | 5'- TGGCTTCTGGTGGAAAACCTCC-3' |
| CCNB1 | 5' – AGAGGTGGAACTTGCTGAGCCT-3' | 5'-GCACATCCAGATGTTTCCATCGG-3' |
| C2cd4B | 5'- GCACCGTTTACGGCTTCTG - 3' | 5' – GGTGGGGTTTTGTCCAGGTC – 3' |
| CDK5 | 5' – GTACTCCACGTCCATCGACATG-3' | 5'- GCCATTGTTCCTCAGTCGGTGT-3' |
| FBP1 | 5'- TGCTGAAGTCGTCCTACGCTAC-3' | 5'- TTCCGATGGACACAAGGCAGTC-3' |
| Acsl4 | 5'-CCTTTGGCTCATGTCTGGAAC-3' | 5'- GCCATAAGTGTGGGTTTCAGTAC-3' |
| Per2 | 5'-ATGACAGAGGCAGAGCACAA-3' | 5'-CAGCTGGTAGTACTCCTCATTAG-3' |

**Supplementary Table 2:**

| Sample | Total Reads | Mapped (%) | Singletons (%) | Mapped Reads | Singleton Reads |
|---|---|---|---|---|---|
| **1Bmal1** | 1.64E+08 | 70.46 | 15.11 | 115298575 | 24715927 |
| **2Bmal1** | 2.16E+08 | 74.05 | 14.01 | 160016040 | 30278944 |
| **3Bmal1** | 2.06E+08 | 73.56 | 13.71 | 151475094 | 28235558 |
| **tdTom1** | 2.06E+08 | 73.41 | 12.12 | 150857367 | 24917629 |
| **tdTom2** | 1.88E+08 | 73.67 | 12.45 | 138557837 | 23413361 |
| **tdTom3** | 2.32E+08 | 72.42 | 14.04 | 167743392 | 32522082 |

**Supplementary Table 2. RNA-Seq alignment summary for samples**.

This table summarizes sequencing alignment statistics for RNA-seq libraries aligned to the mouse genome (mm10). Total Reads indicates the number of paired ends reads obtained per sample. Mapped (%) represents the percentage of reads successfully aligned to the reference genome. Mapping efficiency was consistent across all samples, with 70–74% of reads successfully aligned. Singletons (%) indicates the percentage of reads where only one end of the pair mapped, singleton reads ranged from 12–15%, indicating minimal technical variability across libraries. These results support the overall quality and comparability of the libraries included in the differential expression analysis. All samples passed standard quality thresholds, and no technical issues were identified for any sample, including the tdTom1 sample that appeared as an outlier in PCA space (Fig. 3B).
